# Dynamic cardiac anatomy: the "cypress tree" papillary muscle root 

**DOI:** 10.15171/jcvtr.2018.22

**Published:** 2018-09-30

**Authors:** Muhammad Shoaib Khan, Robert Biederman

**Affiliations:** Department of Cardiac MRI, Allegheny General Hospital, Pittsburgh, USA

**Keywords:** Cypress Roots, Papillary Muscle, Contemporary Papillary Muscle Model, Classic Papillary Muscle Model, CMR

## Abstract

***Introduction:*** The understanding of gross cardiac anatomy has been relatively stable over the last 80 years, reliant on well-established autopsy findings. The advent of dynamic imaging by cardiac MRI and CT provides a window to view anatomic features in vivo, providing insights typically masked at autopsy due to death.

***Hypothesis:*** We hypothesize that cardiac magnetic resonance (CMR) with its high spatial and temporal resolution allows detection of anatomic features not previously appreciated at autopsy.

***Methods:*** Two hundred fifty-five sequential, CMR examinations were retrospectively examined to describe the anatomic features of the LV (left ventricular) PM (papillary muscles). Specifically, the origin of the base of the PM was delineated. The insertion of the PM was seen in 255/255 patients.

***Results:*** In 249 out of 255 patients (97.6%), the appearance of the PM was not a uniform muscle arising from the inner face of the LV myocardium, but was a finger-like series of long, slender trabeculae carneae traversing >1 cm before inserting into the main body of PM challenging our previous understanding of PM anatomy.

***Conclusion:*** The capabilities of dynamic CMR to view cardiac features in vivo non-invasively provides a useful tool to study cardiac anatomy. Unlike the widely accepted representation of papillary muscles, uniformly arising from the floor of the LV, the base resolves into a ‘cypress-tree’ root-like structure with multiple thin projections before coalescing into a thick muscle head. Such observations have far reaching clinical implications in areas such as mitral regurgitation, post-MI remodeling and electrical transmission of the His-Purkinje system, and further work is indicated to delineate the role of non-invasive imaging in these areas.

## Introduction

In the last few decades, our understanding of the gross anatomy of the heart has been quite static. Predominant gross anatomy literature is based on the findings from the autopsied heart. However, with the emergence of dynamic imaging utilizing cardiac magnetic resonance (CMR), there has been a renewed opportunity to visualize cardiac anatomy features *in vivo*, which historically has not been apparent on gross anatomy of the autopsied hearts.

**Figure 1:**
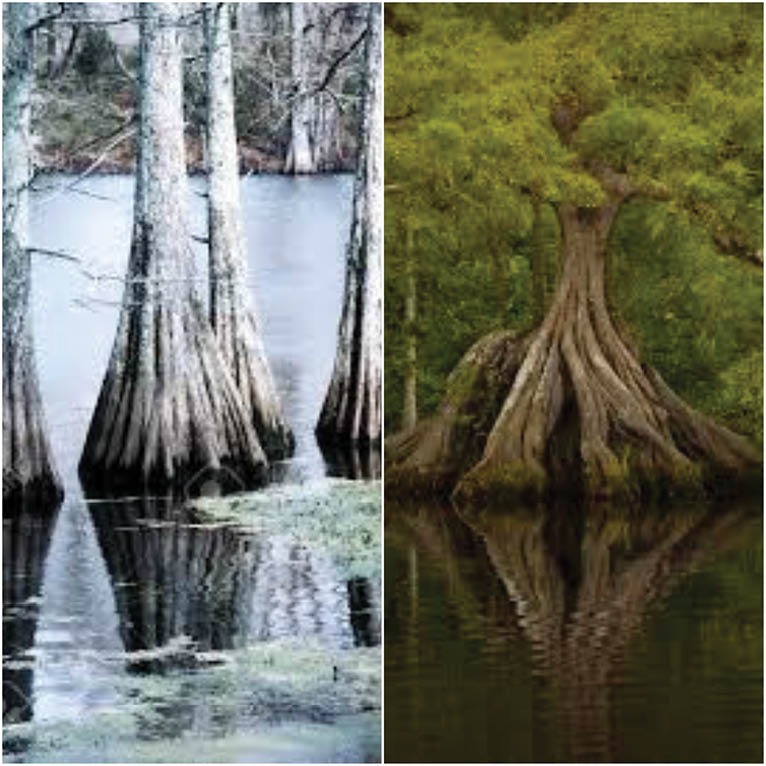
Cypress Tree Roots: Note the multiple roots arise off from the floor of the swamp as separate and distinct roots but eventually coalesce to form the trunk of the cypress tree.

Using advanced imaging with CMR, we have observed an intriguing pattern of papillary muscle (PM) attachments in which the muscle appears to take origin from the ventricular myocardium as a series of entwined tentacles which travel a distance of more than 1 cm before merging to form the main PM body, from the tip of which arise chordae tendineae. These PM tentacles bear resemblance to the roots of cypress tree (Figure 1), which arise off from the floor of the swamp as separate and distinct roots but eventually coalesce to form the trunk of the cypress tree. Via CMR, these tentacles of PM are more obvious during diastole and less/not obvious during systole potentially explanatory for their unrecognized state to date. We have herein named this unique model of PM attachment, based on the CMR findings, as the “contemporary model” of PM anatomy.

This “contemporary model” of ours, we propose, is in contrast to the “classic model” of PM attachments, wherein the PM is thought to originate from the ventricular myocardium as a uniform muscle rather than as a series of tentacles.

## Hypothesis

The above mentioned contemporary model of PM, as seen on CMR imaging, which reveals perspicuously during diastole and conceals fleetingly during systole has led us to hypothesize that this kind of model is overlooked on gross anatomy perhaps because the heart is in a contracture (systolic) state after the death masking its’ formation. Similarly, in the operative suite, such is not recognized during induced cardiac arrest. However, CMR with its high spatial and temporal resolution allows *in-vivo* appreciation of such a model, which has not been evidently described before based on autopsy findings.

## Materials and Methods

**Figure 2:**
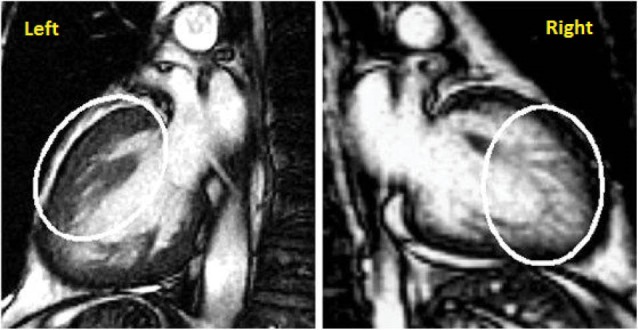
Long axis view of the heart on MRI: Classic model of PM seen during systole (left). Contemporary model of PM seen during diastole (Right). During diastole, origin of PM from ventricular wall is seen as tentacles (in gray within encircled area), which coalesce to form main papillary muscle body (small, dark conical area protruding outside the top left of the white circle).

All patients underwent imaging on a 1.5 T GE (GE-CVI Excite version 12, Milwaukee, WI) CMR scanner. CMR scan protocols included: cine-CMR imaging involving cardiac structure and function assessment. All cardiac images were obtained using multiple breath hold, ECG gated, steady-state free precession (SSFP) technique (typical parameters included were slice thickness 8 mm, field of view depended on body habitus, matrix size 224 x 224, echo time 1.2 ms, repetition time 2.9 ms, and flip angle 45°, NEX 0.75). In patients who could not co-operate with breath holds, we adjusted the NEX, phase and number of views per segment in order to improve image quality. Gadolinium was not utilized for the determination of the papillary origins. Imaging for this study was typically performed in the 2, 3 and 4-chamber views. Analysis was performed on a standard platform utilizing the GE Workstation, a commercial analysis platform without modification. Interpretation was performed by both of the CMR readers, with one reader (Robert Biederman) having over 20 years of experience. Interrogation was performed on the representative images as to the origin of the PM with relation to the insertion into the left ventricular endocardium/myocardium. If the insertion into the LV wall (endocardium) was broad and continuous over the PM base without interruption or discontinuity and no blood pool was present and no interdigitation was visible on high resolution CMR, then the PM was classified as ‘classic’ referring to the traditional/accepted and most recognized typical understanding of the papillary/ left ventricle (LV) insertion. Similarly, if the papillary inserted into LV endocardium/myocardium as a series (>1) of well-defined long, linear root-like, finger-like projections with clear, unobstructed and unequivocal blood pool interdigitating between such structures, then the PM was classified as ‘contemporary’ representing the phrase ‘Cypress Root’ (Figure 2). If the cypress root formation was visible in any or all of the 3 CMR projections (2, 3 or 4-chamber), it was confirmed as such. Alternatively, if no such configuration was present in any of the 3 projections the term was not utilized.

## Results

Consecutively, without selection for gender, age or disease state, 255 sequential, CMR examinations were retrospectively examined to describe the anatomic features of the LV PMs. No complications occurred. No additional time was required for performance of the SSFP acquisition for the determination of the papillary structures. Average time per breath hold sequence representing 2 slices per projection (6 total) was 8 minutes. Average time for reader interrogation per patient was <60 seconds and many times, upon the learned recognition was immediately apparent. Estimated learning curve reduced the time from ~60 seconds to <5 seconds upon repetition and learned behavior.

The population was deemed representative of a typical clinical cohort likely to be referred to a tertiary CMR Center in Pittsburgh, PA. The cohort represented 66% male (64±7 years) 36% female (68±6 years) (*P*<0.05 for male/female) with variable diagnostic presentations: HTN; 8%, HCM; 9%, CHD; 17%, PH/PAH; 18%, AF; 13%, CMX; 21%, and miscellaneous; 14%. There was no statistical difference between gender or presentation or frequency within groups.

**Figure 3:**
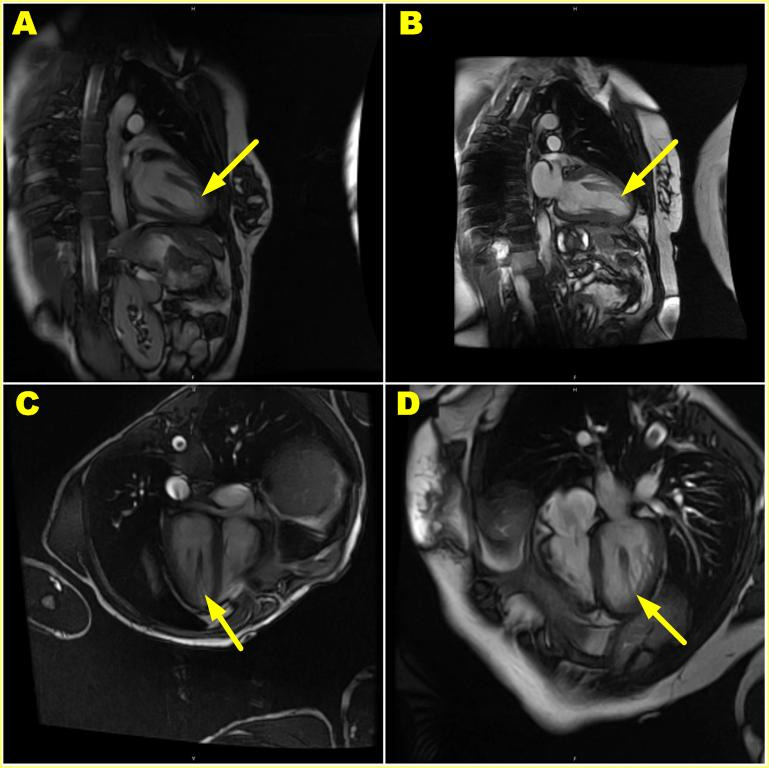
Upper panel (A and B) shows two chamber views of the heart on MRI (SSFP). Lower panel (C and D) depicts four chambers of the heart on MRI (SSFP). Tentacles of papillary muscles (indicated by yellow arrow), bearing resemblance with that of “Cypress roots”, coalesce to form the main body of papillary muscle

CMR image quality was judged exceptional in >98% of patients. Specifically, as described, the origin of the base of the PM was delineated. The insertion of the PM was present and seen in 255/255 patients. In 249 out of 255 patients (97.6%), the appearance of the PM was not a uniform muscle arising from the inner face/endocardium of the LV myocardium, but instead was a finger-like series of long, slender trabeculae carnae traversing >1cm before variably inserting into the main body of PM denoting a cypress root formation (Figure 3); a contemporary finding. As the term ‘parachute’ has been inculcated to represent the mitral/chordae tendinae apparatus, so to the term ‘upside down parachute’ may be taught to define the cypress root/LV endocardial interface. In the remaining 6/255 (2.4%) a cypress root formation was either not found, not present in 3/3 projections or was deemed to be a broad-based contiguous insertion representing the classical representation. In none of the patients in whom image quality was not ‘exceptional’ was a cypress root formation not seen. Likewise, disease state had no influence on the delineation of the classical structure.

## Discussion

### Brief anatomy of papillary muscles

Usually, there are three PMs in the right ventricle^1,2^: a large anterior PM arising from the anterior wall of the right ventricle; a large posterior PM arising from inferior wall of the right ventricle; a small septal PM attached to the Inter-ventricular septum^1^. Whereas, there are two PMs in the LV^3^: The anterolateral muscle arising from the sternocostal wall of the ventricle; the posteromedial muscle arising from the diaphragmatic wall near its anterior end.^3^

The anterolateral PM blood supply is from the left anterior descending (LAD) artery and the diagonal or a marginal branch of the circumflex artery. Depending upon the dominant blood supply, right coronary artery (RCA) or left circumflex artery supplies blood to the posteromedial PM.^4^ As a result of dual blood supply of anterolateral PM, it’s somewhat more protected from ischemic injuries compared to the posteromedial PM, which has only one source of blood supply.^2^

PM functionality is influenced by both the sympathetic and parasympathetic fibres. The heart receives innervation from the cardiac plexus, which itself receives fibers from both the divisions of autonomic nervous system. Fibers from the cardiac plexus supply the SA node and signal from the SA node is conducted to the AV node by the cardiac muscle. The AV bundle carries the signal from the AV node and then divides into right and left bundles that pass on either side of the muscular interventricular septum. These fibres of the right and left bundles, so called purkinje fibres, spread into the walls and PMs of the ventricles. ^5^

### Classic versus Contemporary Model of Papillary Muscle

The origin of PM from the ventricular myocardium as an integrated series of tentacles challenges our previous understanding of PM anatomy. In classical description of PM anatomy, these PM are mainly thought to have their origin as conical or finger like muscular projections arising uniformly from the ventricular wall,^1,4^ and muscular trabeculations firmly fix the PMs to the underlying LV wall.^4^ Tendinous cords arise from the PM and attach to the free edges and ventricular surfaces of AV cusps, much like the suspension lines of a parachute.^1^

In contrast to this classic PM anatomy model, our “contemporary” model of papillary anatomy based on the findings of dynamic CMR, describes PM to have its origin from ventricular wall as a series of tentacles or roots, much like the roots of a cypress tree (Figure 1), which travel a distance of over one cm before merging to form the main body of PM, from which arise the tendinous cords and which then attach to the AV valve cusps in a manner described earlier. This “contemporary” model of PM, gives rise to a picture of valvular apparatus akin to two parachutes lined in the vertical direction, having a common basket and their canopies facing in the opposite direction. PM tentacles (as described earlier) and tendinous cords represent the suspension lines of the two parachutes, valvular cusps (in closed state) and cypress root/LV endocardial interface represent canopies of the two parachutes, and PM body represents the common basket of those two vertically opposing parachutes. This picture is in contrast to the “classic PM model”, which unifies a part of single parachute/umbrella (when cusps are closed) whereby the canopy is formed by the cusps, suspension lines by the tendinous cords and basket is formed by the PM body and underlying myocardium.

### Why has contemporary model not been appreciated before on gross anatomy?

The heart, as a muscle, is affected by the rigor mortis as well, which causes contraction of the heart. One study demonstrated that the heart wall becomes thicker post mortem (as a result of contraction), which is consistent with the knowledge in forensic medicine and pathology that the ventricular walls appear hypertrophic after death.^6^ This could be one possible reason why such PM anatomy has never been explicitly documented before in the traditional gross anatomy literature since the heart dies in a contracted state (systole) and, therefore, gross anatomy of the autopsied heart doesn’t allow appreciation of such tentacles of PM, which are more evident during diastole and almost disappear during systole. The similar notion applies during CABG procedure when the heart is arrested in a depolarized state by potassium solution.^7,8^

### Comparison of different imaging techniques for in-vivo study of papillary muscle anatomy

Such an exquisite PM anatomy, demonstrating “tentacles” originating from the ventricular myocardium before coalescing to form PM body as witnessed on CMR, is also comparably seen on cardiac CT scans as current CT scanners have a spatial resolution of 0.5–0.625 mm in the z-axis (better than that of MRI), and approximately 0.5 mm in the x- to y-axes.^9^ In one of the prior studies,^10^ Leon Axel noted the nature and location of the attachment of the PM to the heart wall via 3D image data acquired using MDCT with standard methods. In all of the cases examined retrospectively (vs. ours; 98%), the base of the PM was seen to not directly join the solid portion of the heart wall. Instead, the base of the PM was seen to be contiguous with the mesh of *trabeculae carneae* lining the ventricular cavity, above the actual surface of the solid part of the heart wall.^10^ This finding seems to be in concordance with that of ours based on CMR imaging. In echocardiography, resolution along the beam direction is typically 1 mm, but resolution across the beam is poor^10^ and also its relatively low contrast resolution does not help appreciating the complexity of the cypress formation. In addition, a natural predilection to not meticulously observe normal anatomical features while performing cardiac echocardiography for pathological indications may be another reason for overlooking such anatomy of PM. Other imaging modalities including PET scan and SPECT, although have very high contrast resolution but their low spatial resolution, 4-15 (FWHM) mm^9^ obfuscates their recognition of the “contemporary” model of PM. Lastly, via catheter angiography for usual indications we see PM as filling defects in ventricles despite the ability of catheter angiography to offer very high spatial resolution [0.16 (FWHM) mm] and a moderate contrast resolution.^9,11^ This is mainly because on catheter angiography to visualize the cardiac muscles including PM, it would require us to perform cine CT imaging of the heart after giving contrast and that would mean unnecessary exposure of the patient to a very high dose of radiations. A comparison of PM structure as seen on different imaging modalities and described in literature before is shown in Table 1.

**Table 1:** Prior literature

**Image Tool**	**Classic Model**	**Contemporary Model**	**Undefined**
Gross Anatomy	(**1**) (**3**)* (**4**) (**18**)* (**19**)*	(**20**) Papillary muscle comes very close to the contemporary model of PM. However, it is quoted from an anatomy atlas that only depicts the model in the picture and does not describe that explicitly	**-**
CMR	Current work	Cypress tree roots	** -**
CT Heart	**-**	(**10**)†describes PM model similar to contemporary one but has not explicitly used the term ‘cypress roots’	** -**
Echocardiography	** -**	**-**	Low spatial resolution
PET	**-**	**-**	Neither model is appreciated likely due to the low spatial resolution
SPECT	**-**	**-**	Neiter model is appreciated likely due to the low spatial resolution
Coronary Angiogram		**-**	Not appreciated on typical LV ventriculogram.

A comparison of anatomy as demonstrated across distinct gross anatomy and imaging technique literature. “Undefined” column does not describe either conventional or contemporary model remaining unspecified in the published literature. Numerical numbers in the table correspond to the appropriate reference. * Literature does not specifically describe either conventional or contemporary model. However, it can be deduced from the images and text that these resources have highlighted a model similar to the classic one. † Describes the papillary muscle body to not have direct attachment with the ventricular wall and the author has apparently described the contemporary model of PM, as have we done.

### Clinical implications of Cypress

Our study demonstrated such pattern of PM model in over 97% of the patients regardless of the fact if the heart was diseased or not. Such contemporary model can have potentially intriguing implications, ranging from physiological to pathological, involving mitral apparatus and even adjoining parts of the heart. Based on the above observations of the “contemporary model” of PM as a cypress root, we can surmise a few clinical implications. There are two forces ensuring fitting closure of atrial fibrillation (AV) valves during systole: (*a*) rising ventricular/atrium pressure gradient^2^ and (*b*) the pull of PM. It was observed in studies that there was a slight delay in the spread of electrical activity from ventricular myocardium to PM body.^10-13^ This delay of impulse conduction is possibly explained by the now-expected additional time taken by electrical impulse to travel through cypress root of the PM and thence to the main body of PM. This slight delay of impulse probably makes sure that the rising ventricular pressure initiates coaptation of valvular leaflets and later the pull of the PM completes and maintains this line of coaptation during systole. Simultaneous initiation of both of these forces would perhaps have made the closure of leaflets difficult. Normal integrity of valve function is maintained by all the components of mitral apparatus, including fibromuscular annulus, appropriate coaptation of mitral leaflets, tendinous cords, PM, underlying myocardium.^14^ Under normal circumstances, the net force spanning across these valvular components favors closure of these AV valves during systole, and opening during diastole. This balance is altered when any of the components of valve apparatus is diseased and hence resulting in inappropriate valve closure.^15^ Such imbalance of forces is proposed to happen during ventricular remodeling as a result of myocardial ischemia/infarction whereby dilation of the heart and increased sphericity tend to displace PM and results in increased tethering length (distance between PM apex and anterior mitral annulus), which leads to incompetent valve.^16,17^ It is proposed that these localized geometric changes may be more important in producing MR compared to global changes in the geometry of heart.^17^ In one of the studies it was found out that in chronic MR after restoring this localized geometry by plicating the infarct and restoring the normal tethering association between PM and mitral annulus, MR either disappeared or reduced.^17^ Our “contemporary model of PM” could prove of paramount importance in guiding therapy for MR patients, as localized restoration of heart geometry is easier as compared to restoration of entire altered geometry of the heart (as occurs in remodeling).

Similarly, arresting the heart in diastole during cardiac valve repair (and not in depolarized state as occurs in conventional open heart surgeries) would allow surgeons to operate on individual PM tentacles, setting the stage for them to vary angulation/position of PM in a manner that would allow reconstruction of appropriate tethering length and hence potentially curing MR.

## Conclusion

Most of our understanding of the heart anatomy is based on the gross examination of the autopsied heart. However, CMR provides us noninvasive imaging tool which can aid us appreciating cardiac anatomy features, in live subjects, that have never been distinctly described in the literature before. The “contemporary model” of PM that we have described, contradicts with the classic model of PM. This insight now provides a new dimension to our understanding of the physiology and pathology of the mitral apparatus, adjoining parts of the heart and a new understanding of a structure thought long understood.

## Funding

This research received no grant from any funding agency in the public, commercial or not-for-profit sectors.

## Ethical approval

The authors declare that there is no conflict of interest.

## Competing interests

All authors declare no competing financial interests exist.

## Acknowledgements

We wish to thank Leon Axel (M.D, PhD) for the many conversations over the years, which have profoundly assisted us in our study.
